# London Lancet

**Published:** 1865-01

**Authors:** 


LONDON LANCET:—This standard representative of English medical literature appears promptly every month, from the Republication Office, 109 Nassau Street, New York.

